# Dand: Reply

**Published:** 2009

**Authors:** Prabha S. Yadav, Quazi G. Ahmad, Vinay K. Shankhdhar, G. I. Nambi

**Affiliations:** Plastic and Reconstructive Services, Department of Surgical Oncology, Tata Memorial Hospital, E. Borges Road, Parel, Mumbai – 400 012, India

**Figure 1:**
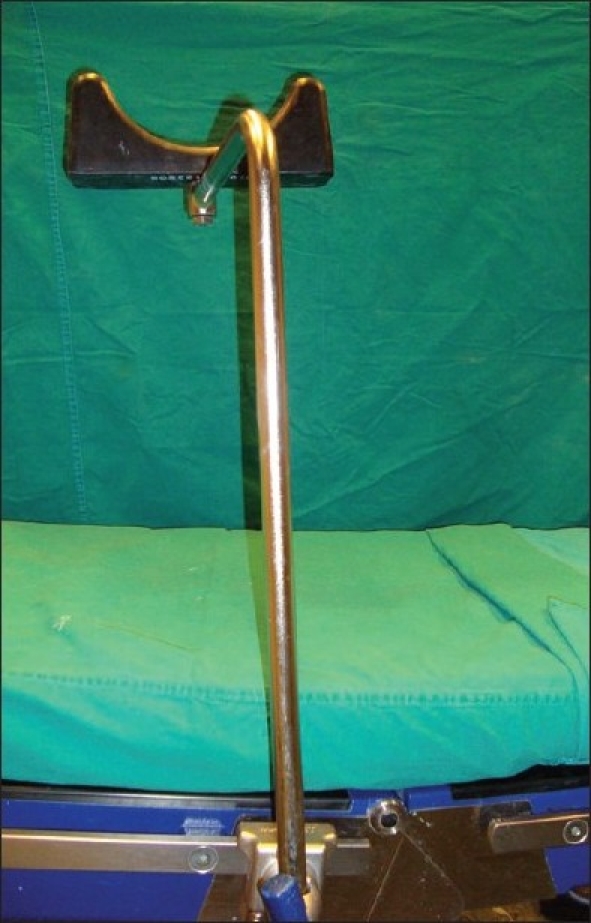
The top of the leg rest which resembles the top of the Dand

**Figure 2:**
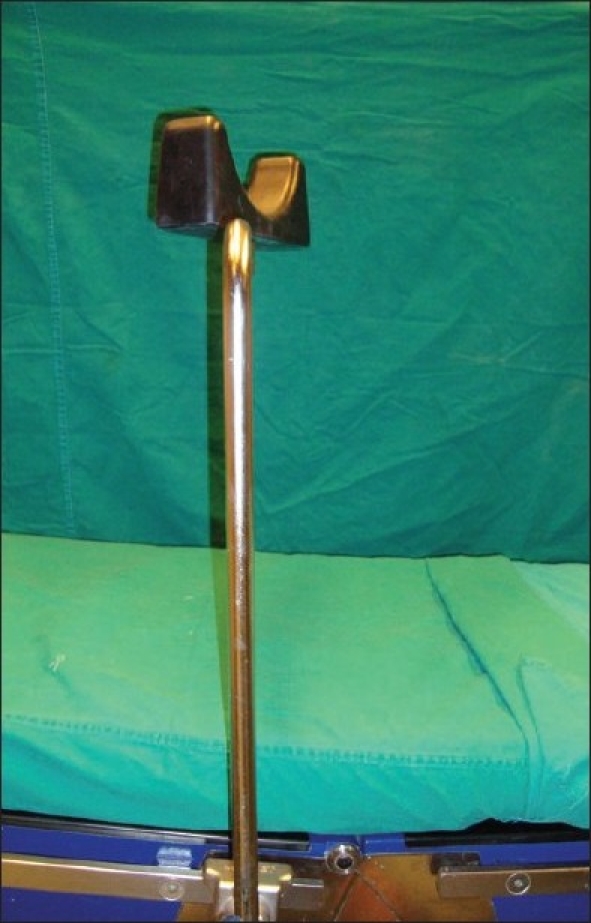
The leg rest mounted on the table and the top can be rotated 360*

**Figure 3:**
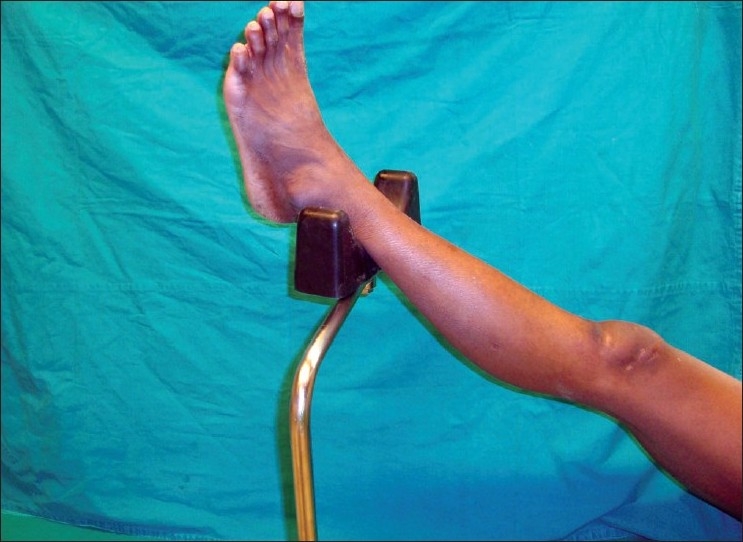
The leg placed on the leg rest during pre surgical scrubbing. The top of the leg rest has a cushion which minimizes pressure effect during long surgeries

Sir,

We congratulate the authors of Dand[1] for their innovative idea and technique, which saves time, man power, energy and reduces the stress of the assistant during the preparation and surgery of the lower limb. However, we would like to share our technique, which is more simple and flexible, for the same procedures.. We use the leg rest which is a part of a standard operating table [Figures 1 and 2] to position the lower limb during scrubbing [Figure 3] as well as in surgeries from upper leg to the foot, which require limb elevation. The sterile environment can be maintained by covering the leg rest with sterile sheets after scrubbing and painting.

The ‘U’ shaped top of the leg rest which in a way resembles the top of Dand is mobile and can be adjusted in any direction. The height of the leg rest can also be adjusted by sliding it up or down through its fixator, which is attached to the handle along the side of the operating table. The top of the leg rest has a cushion which minimizes the pressure effect during long surgeries and two leg rests can be used if both the lower limbs require preparation simultaneously. We conclude that though Dand is innovative, the above factors make the leg rest more flexible than the Dand with all the advantages of Dand.
